# 14-3-3 Proteins are Regulators of Autophagy

**DOI:** 10.3390/cells1040754

**Published:** 2012-10-15

**Authors:** Mercedes Pozuelo-Rubio

**Affiliations:** Centro Andaluz de Biología Molecular y Medicina Regenerativa, Consejo Superior de Investigaciones Científicas. Av. Américo Vespucio s/n, Sevilla-41092, Spain; Email: merce_pozo@yahoo.es; Tel.: +34-600826730; Fax: +34-954461664

**Keywords:** 14-3-3 proteins, autophagy, cell signaling

## Abstract

14-3-3 proteins are implicated in the regulation of proteins involved in a variety of signaling pathways. 14-3-3-dependent protein regulation occurs through phosphorylation-dependent binding that results, in many cases, in the release of survival signals in cells. Autophagy is a cell digestion process that contributes to overcoming nutrient deprivation and is initiated under stress conditions. However, whether autophagy is a cell survival or cell death mechanism remains under discussion and may depend on context. Nevertheless, autophagy is a cellular process that determines cell fate and is tightly regulated by different signaling pathways, some of which, for example MAPK, PI3K and mTOR, are tightly regulated by 14-3-3 proteins. It is therefore important to understand the role of 14-3-3 protein in modulating the autophagic process. Within this context, direct binding of 14-3-3 to mTOR regulatory proteins, such as TSC2 and PRAS40, connects 14-3-3 with autophagy regulatory processes. In addition, 14-3-3 binding to human vacuolar protein sorting 34 (hVps34), a class III phosphatidylinositol-3-kinase (PI3KC3), indicates the involvement of 14-3-3 proteins in regulating autophagosome formation. hVps34 is involved in vesicle trafficking processes such as autophagy, and its activation is needed for initiation of autophagy. Chromatography and overlay techniques suggest that hVps34 directly interacts with 14-3-3 proteins under physiological conditions, thereby maintaining hVps34 in an inactive state. In contrast, nutrient starvation promotes dissociation of the 14-3-3–hVps34 complex, thereby enhancing hVps34 lipid kinase activity. Thus, 14-3-3 proteins are regulators of autophagy through regulating key components of the autophagic machinery. This review summarizes the role of 14-3-3 protein in the control of target proteins involved in regulating the master switches of autophagy.

## 1. Introduction

### 1.1. Overview of 14-3-3 Proteins

The cellular decision to live or die is finely controlled by tightly regulated signaling pathways. 14-3-3 proteins have a key role in this decision by controlling many of the signaling pathways that mediate this process.

14-3-3 proteins comprise a large family of acidic proteins that are expressed within all eukaryotic cells and function as homodimers and heterodimers [1,2]. They perform key regulatory roles by binding to and modulating the function of target proteins [3], principally through interacting with specific phosphoserine and phosphothreonine motifs [4]. Phosphorylation-dependent association with binding partners forms the mechanistic basis for the fundamental role for 14-3-3 in modulating kinase signaling pathways. A large number of 14-3-3 binding motifs have been established [5], and these have recently been reviewed [6]. Seven mammalian 14-3-3 isoforms have been reported (α/β, ε, γ, σ, ζ/δ, τ/θ and η) that are encoded by seven different genes and vary in their expression levels between tissues [7,8,9]. Moreover, highly redundant functions exist between the different 14-3-3 isoforms. Nevertheless, the 14–3-3σ isoform has distinct structural properties that principally promote the formation of homodimers, and this isoform is mainly associated with regulating cell proliferation. 14-3-3 binding can alter the enzymatic activity, subcellular localization, protein-protein interactions, phosphorylation status and proteolysis of target proteins [10]. Many 14-3-3 target proteins are deregulated in human diseases such as cancer, diabetes, Parkinson’s disease and other neurological diseases [11]. Furthermore, proteomics analysis suggests that 14-3-3 proteins are central regulators of different biological processes, including cell signaling, cell cycle progression, cytoskeletal dynamics, cell metabolism and making the decision between cell death and survival [12,13,14,15,16,17,18,19].

### 1.2. Role of 14-3-3 in Apoptosis

The role of 14-3-3 in apoptosis has been well documented and several reports indicate that 14-3-3 protein interaction with target binding partners initiates events that support cell survival, thus mediating an essential anti-apoptotic signal [20]. It has been reported that 14-3-3 proteins bind to members of the Bcl-2 family, Bcl-xL⁄Bcl-2-associated death promoter (BAD) and Bcl-2-associated X protein (BAX), thereby inhibiting their proapoptotic activities [21,22]. Additionally, 14-3-3 overexpression blocks cell death initiated by other death promoters, such as apoptosis signal-regulating kinase 1 (ASK1) [23]. Moreover, 14-3-3 protein interaction with a member of the forkhead family of transcription factors, forkhead box protein (Fox03a), blocks nuclear translocation and the transcription of death genes [24]. Thus, 14-3-3-dependent apoptosis suppression through association with ASK1, BAD and Fox03a suggests that 14-3-3 has a key anti-apoptotic function in cells. Furthermore, expression of a polypeptide that blocks 14-3-3 association with binding proteins promotes apoptosis and reduces viability in several cancer cell lines [25,26]. The use of 14-3-3 ζ antisense RNA molecules in cancer cell lines increases sensitivity to stress-induced apoptosis [27,28,29]. In addition, cell treatment with 2-methoxyestradiol results in reduced 14-3-3 expression that promotes apoptosis activation and inhibits cell growth [30]. A recent comprehensive proteomics analysis of 14-3-3-binding proteins in unchallenged cells, compared with those subject to an apoptosis stimulus, suggests new cell survival functions for 14-3-3 proteins [16]. Taking into account the finding that 14-3-3 protein association with binding partners induces a cell survival signal [20], it is of great interest to analyze the role of 14-3-3 protein in a mechanism that controls cell fate, such as autophagy.

### 1.3. Autophagy Process Summary

Macroautophagy (hereafter referred to as autophagy) is a self-digestion process that is enhanced by several cancer-related stimuli, as well as starvation, TNFα and ceramide. Autophagy may promote cell adaptation to starvation conditions produced by reduced extracellular or intracellular nutrients as a consequence of loss of growth factor signaling. Autophagy is characterized by the engulfment of the cell cytoplasm and organelles in double membrane vesicles, or autophagosomes, resulting in their degradation [31]. Autophagic degradation products are used as source materials for metabolic processes that enable the cell to obtain sufficient energy in order to survive under conditions of cellular stress and nutrient deprivation [32]. Under normal growth conditions, autophagy is a highly regulated process involved in the turnover of long-lived proteins or in eliminating damaged organelles [33,34]. It is notable that excessive autophagy may promote cell death [35].

The autophagic machinery includes groups of proteins that control autophagosome formation and thus the rate of degradation of sequestered material and cellular energy levels. Autophagosomes fuse with lysosomes to form autolysosomes, in which cellular components are degraded by lysosomal hydrolases [36] through a series of steps (reviewed in [37]). In yeast, the induction of autophagy involves inhibition of serine/threonine kinase TOR (target of rapamycin), which under normal growth condition blocks autophagy by constitutive phosphorylation of autophagy protein-13 (Atg13). Following TOR inhibition, Atg13 forms a protein complex containing Atg1 kinase and Atg17, which induces membrane isolation. The mammalian homologues of Atg1 are ULK1 and ULK2 (Unc-51-like kinase 1 and 2) and the Atg17 homologue is FIP200 (focal adhesion kinase family-interacting protein of 200 kDa) [38]. The ULKs and FIP200 form a complex with mammalian Atg13 that translocates to the phagophore under conditions of starvation and mediates autophagy initiation [39]. Under normal growth conditions, the mammalian homologous of Atg1 is inhibited by mammalian target of rapamycin signaling complex 1 (mTORC1) [40]. In the mammalian system, p62/SQSTM1 recognizes ubiquitinated proteins and binds to LC3 (microtubule-associated protein 1 light chain 3), leading to sequestration of polyubiquitinated proteins within the autophagosome and their subsequent degradation [41,42]. The subsequent activation of mammalian Vps34, a class III phosphatidylinositol 3-kinase (PI3KC3), generates phosphatidylinositol-3-phosphate (PtdIns3P) and initiates vesicle nucleation. Vps34 activation is dependent on the formation of a multiprotein complex involving Beclin-1, UVRAG (UV irradiation resistance-associated tumor suppressor gene), a myristylated kinase p150 and Atg14L. Vesicle elongation involves two ubiquitin-like conjugation systems. Thus, the conjugation of phosphatidylethanolamine to LC3 transforms the soluble form of LC3 (named LC3-I) to the autophagic vesicle-binding form (LC3-II). GFP-tagged LC3-II can be used as a marker of autophagy induction, as membrane association promotes a shift from a diffuse to a punctuate fluorescent signal. Autophagosomes mature by fusion with lysosomes to form autolysosomes that degrade the contents of the autophagic vacuoles using lysosomal enzymes [37].

Under different experimental conditions, autophagy has been reported to constitute a stress adaptation to avoid cell death, a failed attempt to rescue stressed cells from death or an alternative cell death pathway [36,43,44,45,46,47]. Whether autophagy is a survival strategy, a death mechanism or both is still being debated: autophagy is essential for maintaining cell survival under conditions of nutrient and growth factor deprivation, but C2-ceramide-induced autophagy may be a form of caspase-independent cell death [48]. Within this dichotomy, determining the role of 14-3-3 proteins in autophagy regulation is of key interest.

14-3-3 proteins form a ubiquitous eukaryotic adaptor protein family involved in numerous cell biology processes, including those related to cell survival or death decisions. This review summarizes the involvement of 14-3-3 family members in cell fate, and emphasizes their recently discovered regulatory roles in autophagy.

## 2. Role of 14-3-3 Proteins in Autophagy

### 2.1. Regulation of TCS2 by 14-3-3 Proteins

**Figure 1:**
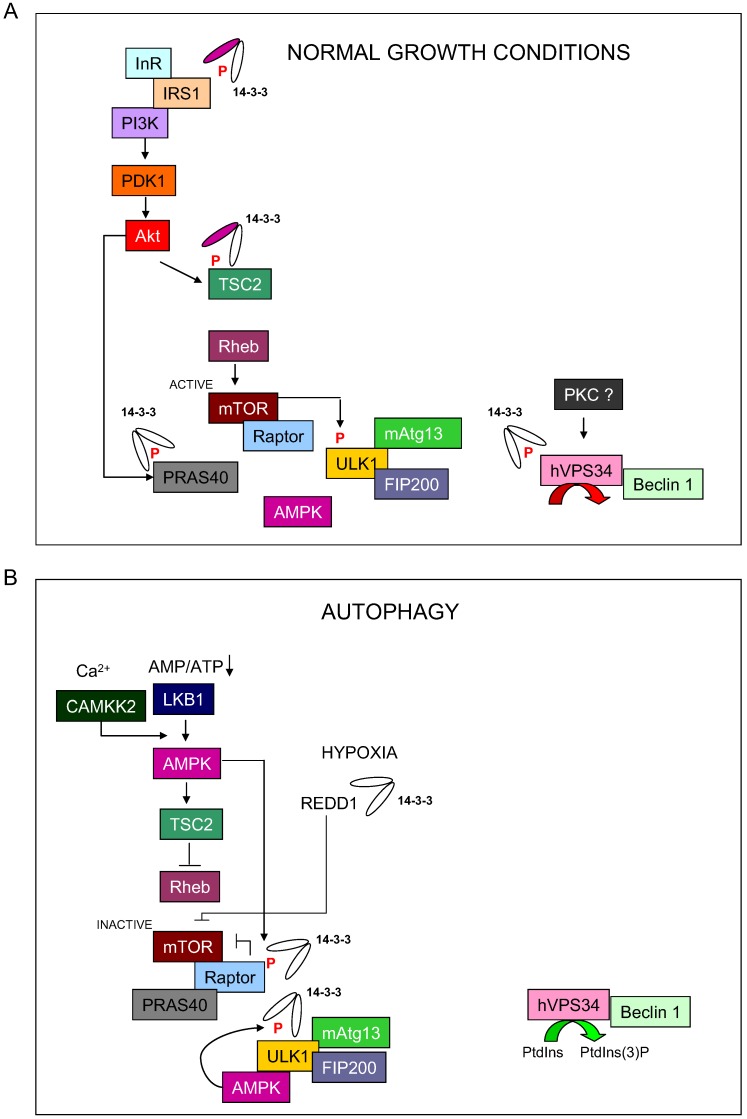
Model of 14-3-3 regulation on autophagy initiation process. (A) During normal growth conditions, 14-3-3 proteins bind and regulate TCS2, PRAS40 and hVps34 to block autophagy. (B) During nutrient starvation, 14-3-3 proteins bind to Raptor and ULK1 to promote the initiation of autophagy.

Autophagy is tightly controlled by signaling pathways that sense the energy and nutrient status of cells. The AMP-activated protein kinases (AMPKs) are important cellular sensors of energy levels that function by sensing the AMP:ATP ratio and regulating metabolic pathways. AMPKs are activated by AMP binding to their γ-subunit, leading to phosphorylation of Thr172 within the activation loop of the catalytic α-subunit. This phosphorylation is promoted by an upstream kinase, such as the tumor suppressor liver kinase B1 (LKB1/STK11) [49]. AMPKs respond to low energy stress by suppressing cell growth and biosynthesis, in part through inhibition of the TOR signaling complex 1 (TORC1), an established regulator of autophagy. TORC1 plays a critical role in sensing and responding to low energy conditions by linking nutrient levels with cell fate [50]. TORC1 controls biogenesis and protein translation through phosphorylation of S6 kinase 1 (S6K1) and eIF4E-binding protein 1 (4E-BP1). An important modulator of the mammalian TORC1 (mTORC1) is the GAP-containing protein complex of tuberous sclerosis proteins 1 and 2 (TSC1/hamartin and TSC2/tuberin). The TSC2 tumor suppressor is reported to be phosphorylated by AMPK at conserved serine sites [51,52,53,54]. Through this phosphorylation-dependent mechanism, TSC1/2 inhibit the Rheb GTPase (a Ras homologue enriched in brain), which is an inducer of mTORC1, thus suppressing mTORC1 activation and leading to autophagy initiation [55]. In addition, under normal growth conditions, stimulation of the insulin signaling pathway promotes Akt (protein kinase B) activation by phosphoinositide-dependent kinase-1(PDK1) and the rapamycin-insensitive companion of TOR (RICTOR)-containing TOR complex 2 (TORC2). Following IGF-1 or insulin stimulation, Akt phosphorylate TSC2 protein at Ser939 and Thr1462, leading to 14-3-3 protein association. Further analysis using point mutants indicates that TSC2 phospho-Ser939 is the primary recognition motif for 14-3-3 proteins [56]. However, other reports indicate that TSC2 Ser1210 phosphorylation by MAP-kinase activated protein kinase 2 (MK2) enhances the interaction between TSC2 and 14-3-3 proteins. Additional studies also established that TSC2 localization and its potential to regulate mTOR/S6K are controlled via 14-3-3 binding [57], which suggests an essential role for 14-3-3 in controlling the initial steps of autophagy through regulating the mTORC1 pathway. Thus, once TSC2 is inhibited by this 14-3-3 binding, GTP-bound Rheb can activate the mTORC1 complex, resulting in protein synthesis, cell growth and inhibition of autophagy [58] (Figure 1).

### 2.2. Regulation of PRAS40 by 14-3-3 Proteins

In addition to TSC2, the 14-3-3-binding partner proline-rich Akt substrate 40 (PRAS40) is reported to be essential for mediating mTORC1 signaling and triggering autophagy [59]. PRAS40 is a component of the mTORC1 complex that probably interacts with Raptor, which mediates PI3K pathway regulation of TORC1 signaling under nutrient or serum deprivation or following inhibition of mitochondrial metabolism [60]. Phosphorylation of PRAS40 Thr246 by Akt and PRAS40 Ser183 and Ser221 by mTORC1 promotes 14-3-3 binding to PRAS40 and its dissociation from mTORC1 [61]. Phosphorylation of PRAS40 Thr246 has been reported to facilitate the efficient phosphorylation of PRAS40 on its mTORC1-dependent sites [62]. In addition, Ser221 to Ala mutation reduces 14-3-3 interaction to the same extent as mutation of the Thr246 Akt site. Moreover, Ser221 mutation increases PRAS40 inhibition of mTORC1 [61]. PRAS40 binding to 14-3-3 proteins is blocked by TSC1/2 and stimulated by Rheb in a rapamycin-sensitive manner, suggesting an important role for PRAS40 in regulating mTORC1 [63]. Thus, PRAS40 functions as a negative regulator when bound to mTORC1, and dissociates from mTORC1 in response to insulin stimulation. PRAS40 phosphorylation and 14-3-3-binding therefore mediate the regulation of mTORC1 by nutrient and growth factor availability, leading to initiation of the autophagy process.

### 2.3. Regulation of Raptor by 14-3-3 Proteins

The mTOR binding partner Raptor has been reported to be a direct substrate of AMPK. Raptor phosphorylation by AMPK at two highly conserved serine residues, Ser722 and Ser792, promotes direct binding to 14-3-3, thus leading to inhibition of mTORC1 kinase activity [64]. AMPK phosphorylation of Raptor is thought to lead to changes in the availability of 14-3-3 for PRAS40 binding, which acts to suppress mTOR kinase activity, thus leading to autophagy initiation.

### 2.4. Regulation of REDD by 14-3-3 Proteins

In addition, hypoxic stress suppresses mTOR activity and induces autophagy. REDD1 (Regulated in development and DNA damages responses 1) expression is reported to be highly induced in response to hypoxia [65]. In mammalian cells, REDD1 overexpression inhibits mTORC1 activity, while suppression of REDD1 prevents mTORC1 downregulation in response to hypoxia [66,67]. Regulation of mTORC1 activity in response to hypoxia has been suggested to occur through REDD1-mediated dissociation of the TCS2–14-3-3 complex. 14-3-3 binding to REDD1 is necessary and sufficient to promote TSC2–14-3-3 dissociation and mTORC1 inhibition [68]. Thus, REDD1 specifically blocks PI3K/Akt-induced TSC2–14-3-3 association and promotes mTORC1 inactivation, leading to autophagy initiation under hypoxia conditions.

In addition, 14-3-3 proteins may control mTORC1 through PI3K pathway regulation in response to nutrient and growth factor depletion by direct binding to IRS-1 (insulin receptor substrate-1). 14-3-3 protein interaction with IRS-1 promotes dissociation of the IRS-1–PI3K class I complex during the insulin desensitization process [69].

### 2.5. 14-3-3 Proteins Regulate MAPK Pathway

Mitogen-activated protein kinases (MAPKs) are serine/threonine kinases that respond to signals such as growth factors and stress, leading to MAPK-dependent activation of a signaling cascade comprising of kinases and transcription factors. MAPK family members include ERK, which is activated by growth signals, and p38 and JNK, which are activated in response to various stresses. Extracellular signals promote activation of Ras, which then binds to and activates Raf, leading to phosphorylation and activation of the two ERK isoforms, ERK1 (p44) and ERK2 (p42) [70]. ERK has been proposed to control autophagy, as well as cell proliferation, migration, differentiation and death [71,72]. ERK induction of autophagy occurs in response to anti-tumor/cytotoxic stimuli through modulating Beclin-1 expression [73,74,75,76,77,78]. In contrast, ERK has also been reported to facilitate malignant growth by inhibiting autophagy-associated tumor suppression [79]. Within these contrasting processes, 14-3-3 proteins may function as adaptor proteins to hold Raf-1 in a partially active conformation prior to full activation [80]. For instance, when the N-terminal Raf-1 14-3-3-binding site is mutated, Raf-1 can activate the MAPK pathway, but is unable to induce cell transformation and differentiation [81]. In addition, 14-3-3 binding to PKA-phosphorylated Raf-1 inhibits Raf-1 translocation to the plasma membrane for interaction with its upstream activator Ras [82].

On the other hand, p38 MAPK has been implicated in cell cycle inhibition, the induction of apoptosis and terminal differentiation [83]. However, p38 has also been implicated in tumor progression, with reported roles in inducing cell invasion, angiogenesis and inflammation [83]. Despite this controversy, p38 is also known to regulate autophagy [84]. Induction of autophagy by p38 is reported to involve increased expression of Atg proteins, such as Beclin-1 and Atg5 [85,86,87,88]. However, other studies have linked p38 inhibition to increased Beclin-1 expression and the induction of autophagy [89,90]. It was recently suggested that p38 inhibits autophagy by competing with the transmembrane protein mAtg9 for binding to p38 interacting protein (p38IP), which is required for mAtg19 cycling during autophagy [84,91].

In this context, MAPK/ERK kinase kinase 3 (MEKK3) is activated by autophosphorylation of Ser526. Association between MEKK3 and 14-3-3 seems to be dependent on Ser526 phosphorylation and this interaction prevents dephosphorylation of Ser526 by PP2A [92]. Thus, transfection of cultured fibroblasts with a double mutant form of 14-3-3 ζ (DN-14-3-3-ζ) inhibited serum-stimulated ERK/MAPK activation, but increased the basal activation of JNK1 and p38 MAPK [93].

### 2.6. 14-3-3 Proteins Regulate Protein Kinase C

Protein kinase C (PKC) comprises a family of phospholipid-dependent serine/threonine kinases that regulate diverse cellular functions. On the basis of their requirement for Ca^2+^ and diacylglycerol and their structural characteristics, PKC isoforms involved in distinct roles in signal transduction pathways are classified as conventional (PKCα, βI, βII and γ), novel (PKCδ, ε, η and θ) and atypical (PKCζ and ɩ) [94]. The hypothesis that PKCs may have a role in autophagy originated with the identification of PKCδ as a suppressor of autophagy in pancreatic ductal carcinoma cells [95,96]. In contrast, PKCδ is thought to promote autophagy in rat parotid epithelial cells in response to hypoxic stress [97]. Thus, a controversy exists regarding the role of PKCδ role in determining cell fate, and PKCδ activation is thought to induce or inhibit autophagy depending on cellular context and the potency of the stimulus. PKCθ is also reported to be involved in endoplasmic reticulum (ER) stress-induced autophagy but has no involvement in amino acid starvation-induced autophagy [98]. ER stress inducers lead to calcium-dependent phosphorylation of PKCθ and translocation of PKCθ to LC3-II containing vesicles in the cytoplasm. In contrast, other conventional PKCs isoforms have been proposed to inhibit autophagy induced by starvation or rapamycin in a PI3K-independent manner [99]. Despite this controversy, a regulatory role for PKC isoforms in autophagy has been reported by several groups. On the other hand, 14-3-3 proteins have been reported to regulate PKC activity, although the nature of this effect varies between PKC isoforms: classical PKC isozymes show an approximate twofold activation, PKCδ shows no significant increase in activity, while PKCε is strongly activated by 14-3-3 proteins [100]. In addition, 14-3-3ε has been reported to modulate PKCα activity [101]. Moreover, several other studies have established that an interaction exists between 14-3-3 ζ and PKCs in rat retina, rodent brain and PC12 cells [102,103]. As mentioned above, there are contradictory reports regarding modulation of PKC activity by 14-3-3 proteins, with either inhibition [104,105,106] or activation [107,108] of PKCs, depending on the 14-3-3 and PKC isoforms involved and the cellular conditions.

### 2.7. 14-3-3 Proteins Regulate CaMKKβ

It is interesting to note that under ER stress, induction of autophagy facilitates the removal of unfolded proteins [109]. Among the ER stress-activated kinases, Ca^2+^/calmodulin-dependent kinase kinase-beta (CaMKKβ) binds 14-3-3, which may have important consequences for the regulation of Akt, CaMKI, CaMKIV and ERK signaling pathways [110]. Under ER stress conditions, the combined effect of CaMKKβ activation and the activation of death associated protein kinase (DAPK) and PKR-like ER-regulated kinase (PERK) can lead to upregulation of autophagy-related genes and downregulation of autophagy inhibitors.

## 3. Role of 14-3-3 Proteins during Autophagy Initiation Process

### 3.1. 14-3-3 Proteins Regulate ULK1

Autophagy is a highly regulated degradation process due to the energy cost involved and its important role in determining cell fate, and many different pathways cooperate to regulate this degradation process. In addition, genetic screens in yeast have identified a number of autophagy-related genes, including the serine/threonine protein kinase Atg1 that forms an active complex with Atg13 and Atg17 to regulate the autophagy initiation process [111,112,113]. Under normal physiological conditions, Atg13 is phosphorylated by TOR leading to Atg1–Atg13–Atg17 complex dissociation and inhibition of autophagy [114]. In mammals, the Atg1 kinase homologues ULK1 and ULK2 [115] are involved in starvation- and rapamycin-induced autophagy [39,115,116,117]. AMPK is reported to induce autophagy by directly activating ULK1 through phosphorylation during nutrient starvation. Two independent groups demonstrated that AMPK directly phosphorylates ULK1, although controversy exists regarding the ULK1 phosphorylation sites: Ser317 and Ser777 have been identified by one group and Ser722 and Ser792 by another [118,119]. In any case, phosphorylation at these sites seems to be required for full activation of ULK1 in response to glucose starvation. ULK1 has also been identified as an AMPK-binding protein during starvation, and the association is mediated by a proline–serine rich (PS) domain in ULK1. Further analysis revealed that AMPK-dependent ULK1 Ser555 phosphorylation promotes 14-3-3-binding by ULK1 *in vivo* and *in vitro*. Thus, Ser555 to Ala mutation blocks 14-3-3 binding to ULK1, and *in vivo* ULK1 phosphorylation is inhibited by expression of a dominant-negative AMPK mutant. Although further investigation is required, these data suggest a role for 14-3-3 in regulating a key protein during the autophagy initiation process. Moreover, mTOR has been reported to interact with the ULK1 kinase domain. In addition, formation of a complex between ULK1, mTORC1, and AMPK coincides with 14-3-3–Raptor binding and initiation of autophagy [119]. Under feeding conditions, increased mTOR activity blocks ULK1 activation through Ser757 phosphorylation, leading to ULK1–AMPK complex dissociation and inhibition of autophagy [118]. These results show ULK1 to be an important point of convergence between pathways that control the autophagy initiation process, which mediates feeding signals from mTOR and starvation signals from AMPK. While a role for 14-3-3 in this pathway has been suggested, further investigation is needed to establish the mechanism whereby 14-3-3 controls ULK1.

### 3.2. 14-3-3 Proteins Regulate hVps34 (PI3KC3)

14-3-3 proteins have also been reported to interact with proteins involved in the vesicle nucleation process during autophagy, such as hVps34 (human version of yeast Vps34), the class III phosphatidylinositol-3-kinase (PI3KC3). Phosphoinositide 3-kinases are enzymes that catalyze phosphorylation of the 3ʹ hydroxyl group of the inositol ring in phosphatidylinositol (PtdIns). PI3Ks are classified into three types based on their structure, substrate specificity and functionality [120]. Class I PI3Ks are usually activated by growth factors, leading the production of phosphatidylinositol-3-phosphate PtdIns(3)P, PtdIns(3,4)P2, and PtdIns(3,4,5)P3. These second messengers increase the membrane localization of pleckstrin-homology (PH) domain-containing proteins, such as PDK1 and its substrate Akt, leading to their activation [121]. Class II PI3Ks appear to be related to cell migration and the control of vascular smooth muscle contraction [122,123]. There is only one class III PI3K member, Vps34 (vacuolar protein sorting 34), which has been shown to mediate vesicular trafficking of proteins containing a PtdIns(3)P binding domain, leading to their localization to the endosomal and lysosomal membranes [124]. Vps34 plays an important role in mediating autophagosome formation by promoting the rapid recruitment of proteins with phospholipid-binding domain, such as the WD40-repeat proteins hWIPI-1alpha [125] and hWIPI2 [126], the human orthologues of the yeast autophagy gene Atg18, to the autophagosome structure during the vesicle nucleation process. WIPI-1alpha has been reported to colocalize with LC3 under conditions of nutrient deprivation [125]. Additional data suggests that the Atg18 phospholipid-binding domain binds PtdIns(3)P and is involved in the formation of the pre-autophagosome structure [127]. Furthermore, the presence of WD40 repeats in hWIPI may mediate multiprotein complex formation that may either play a scaffolding role during autophagosome generation or be involved in LC3 lipidation [125,126,128].

hVps34 forms a complex with Beclin-1 that participates in autophagosome formation. Regulatory mechanisms directing Beclin-1/hVps34 specificity were initially shown to involve complex formation with proteins such as Atg14L and Rubicon. Further analysis of these proteins suggested that Atg14L enhances hVps34 lipid kinase activity and upregulates autophagy, whereas Rubicon reduces hVps34 activity and downregulates autophagy [129]. Following initial reports that 14-3-3 proteins may also regulate hVps34 [130], 14-3-3-binding protein affinity purification and DIG-14-3-3 overlay assays revealed that hVps34 directly interacts with 14-3-3 under normal growth condition in a phosphorylation-dependent manner. Additionally, 14-3-3 was established to dissociate from hVps34 during C2-ceramide-induced autophagy, probably in response to protein phosphatase activation during C2-ceramide treatment. Thus, endogenous 14-3-3 and hVps34 proteins interact under normal growth conditions and this association is reversed during autophagy initiation by starvation or treatment with C2-ceramide, rapamycin or etoposide. Furthermore, hVps34 lipid kinase activity is increased following dissociation from 14-3-3 during starvation-induced autophagy or downregulation of 14-3-3 ζ. Moreover, 14-3-3 ζ overexpression decreases hVps34 activity under normal growth conditions. These data suggest that 14-3-3 proteins regulate hVps34 activity, leading to important effects on the autophagy initiation process. As mention above, 14-3-3 regulation of hVps34 seems to be dependent on phosphorylation. Stimulation of cells with the phorbol ester PMA promotes hVps34–14-3-3 binding and decreases hVps34 kinase activity. This effect is dependent on the PKC inhibitor H-7, suggesting that PKC isoforms, or a kinase activated downstream of PKC, induce phosphorylation-dependent hVps34 binding to 14-3-3. Moreover, *in vitro* phosphorylation by PKC promotes hVps34–14-3-3 binding. Additionally, experiments using deletion mutants and site-directed mutagenesis indicate that the N-terminal region of hVps34 mediates 14-3-3 binding and that phosphorylation at several sites may be required for hVps34–14-3-3 binding (Thr197 and Ser212). These data are consistent with the negative regulation of autophagy by PKC and the role of 14-3-3 in negatively regulating both hVps34 and the autophagy initiation process. Data also suggest that 14-3-3 ζ overexpression inhibits autophagy promoted by C2-ceramide or starvation in HeLa and HEK293T cells. Furthermore, depletion of 14-3-3 ζ promotes autophagy in cervix and breast cancer cell lines under normal culture conditions. A significant reduction in Beclin-1 protein expression correlating with positive regulation of autophagy has been observed in U20S cell lines stably expressing inducible or transient siRNA against 14-3-3 τ (θ) [131]. Nevertheless, siRNA-mediated downregulation of 14-3-3 ζ, σ or θ specifically depletes the respective 14-3-3 isoform and induces autophagy, but has no effect on the levels of expression of autophagy-related proteins, included Beclin-1. These data suggest a role for 14-3-3 proteins as negative regulators of autophagy and indicate that this function is mediated by inhibition of hVps34 kinase activity.

## 4. Conclusions

It is becoming clear that autophagy is a tightly regulated process and that this cellular degradation process must only be initiated under specific cellular conditions. 14-3-3 proteins provide an additional level of regulation to this process by binding and thereby controlling the function of key regulatory proteins involved in autophagy. 14-3-3 proteins can directly interact with IRS-1 and this interaction may regulate the ability of the IRS-1 to recruit and activate PI3K, leading to consequences for mTOR regulation and autophagy initiation [69]. Furthermore, 14-3-3 controls autophagy by binding to two regulators of mTOR activation, namely TSC2 and PRAS40 proteins, and probably by association with other autophagy-related proteins that remain to be identified. Under normal growth conditions, 14-3-3 protein binding to TSC2 and PRAS40 leads to mTOR activation. Nutrient or serum deprivation promotes mTOR inactivation through dissociation of the TSC2–14-3-3 and PRAS40–14-3-3 complexes. Moreover, 14-3-3 proteins may also control the autophagy process at a later stage by interacting with and regulating proteins involved in autophagosome formation, such as hVps34. This interaction blocks hVps34 activity under normal growth conditions, while nutrient deprivation promotes 14-3-3–hVps34 dissociation, leading to hVps34 activation. In addition, 14-3-3 proteins may associate with Raptor and ULK1 under conditions of nutrient deprivation and phosphorylation-dependent 14-3-3 association with Raptor may reduce the availability of 14-3-3 for PRAS40 binding, which may have the combined effect of suppressing mTOR kinase activity and inducing autophagy.

More research is required to fully define the role of 14-3-3 in autophagy. However, in addition to having an anti-apoptotic role, 14-3-3 proteins, along with other anti-apoptotic proteins such as Bcl-2 [132] during autophagosome generation and cFLIP during in autophagosome elongation [133], may also regulate cell fate by their anti-autophagic function. Further studies will increase our knowledge about the molecular processes involved in autophagy and may help to increase the efficacy of anti-tumor therapies that target cell fate control.
